# The Characteristics of Chronic Inflammatory Demyelinating Polyneuropathy in Patients with and without Diabetes – An Observational Study

**DOI:** 10.1371/journal.pone.0089344

**Published:** 2014-02-19

**Authors:** Samantha K. Dunnigan, Hamid Ebadi, Ari Breiner, Hans D. Katzberg, Carolina Barnett, Bruce A. Perkins, Vera Bril

**Affiliations:** 1 Division of Neurology, Department of Medicine, University of Toronto, Toronto, Canada; 2 Division of Endocrinology and Metabolism, Department of Medicine, University of Toronto, Toronto, Canada; University of Würzburg, Germany

## Abstract

**Introduction:**

We aimed to determine whether the clinical characteristics and electrodiagnostic classification of nerve injury, and response to treatment differed in patients diagnosed with chronic inflammatory demyelinating polyneuropathy (CIDP) with and without diabetes.

**Methods:**

CIDP patients with diabetes (CIDP+DM) (n = 67) and without diabetes (CIDP-DM) (n = 67) underwent clinical examination and nerve conduction studies (NCS). CIDP-DM patients were selected using age and gender matching with the existing CIDP+DM cohort. Patients treated with immunotherapies were classified as responders (R) (n = 46) or non-responders (NR) (n = 54) based on clinical response to treatment. The groups were compared using analysis of variance, contingency tables and Kruskal-Wallis analyses.

**Results:**

CIDP+DM subjects had more severe neuropathy based on higher lower limb vibration potential thresholds (VPT)(p = 0.004), higher Toronto Clinical Neuropathy Score (TCNS) (p = 0.0009), more proximal weakness (p = 0.03), more gait abnormality (p = 0.03) and more abnormal NCS. CIDP+DM subjects had more abnormal sural NCS with lower sural sensory nerve action potential amplitudes (2.4±3.0 µV, 6.6±6.0 µV, p<0.0001) and slower sural nerve conduction velocities (38.6±5.4 m/s, 41.0±5.3 m/s, p = 0.04). CIDP-DM subjects were more likely to receive immune therapies (93% vs 57%, p = <0.0001), despite no significant differences in treatment responder rates (p = 0.71). Patients who responded to therapy had shorter duration of CIDP than non-responders (8.0±6.0 y vs 11.9±7.6 y, p = 0.004).

**Discussion:**

The clinical phenotype and electrophysiological profile of CIDP patients differs according to the presence or absence of diabetes. Despite CIDP+DM patients having more severe clinical and electrophysiological neuropathy, they are less likely to receive disease-modifying/specific therapy, yet have similar response rates to treatment as those without diabetes. Specifically, the duration of neuropathy - not diabetes status - was associated with treatment response.

## Introduction

Chronic inflammatory demyelinating polyneuropathy (CIDP) is an immune-mediated inflammatory disorder of the peripheral nervous system. Classically, CIDP results in demyelination of peripheral nerves, as indicated by a significant reduction of motor conduction velocity and prolonged distal motor latencies [1], [2]. In brief, electrodiagnostic criteria that may indicate a demyelinating neuropathy are defined for motor nerves and include abnormal distal latency in over 50% of nerves, abnormal motor conduction velocity in over 50% of nerves, or abnormal F wave latency in over 50% of nerves [2], [3]. The diagnosis of CIDP in diabetes patients may be significantly more difficult than in non-diabetics, due to mild demyelinating changes associated with diabetic sensorimotor polyneuropathy (DSP), in the setting of poor glycemic control [4]. Moreover, a number of authors have noted co-existence of CIDP and DSP[5]–[11]. Although no consensus has been reached, some authors have reported lower CMAP amplitudes in CIDP patients with DSP, resulting from dual nerve pathologies [5], [6]. Others have suggested that in diabetes patients, the presence of two electrodiagnostic abnormalities consistent with demyelination supports a diagnosis of CIDP, compared to only one such abnormality for non-diabetes patients [12]. This diagnostic challenge underscores the importance of distinguishing CIDP from DSP in diabetes patients, as the former may be amenable to treatment with immunomodulatory therapies, including intravenous immunoglobulin (IVIg), corticosteroids and plasma exchange, even in the presence of an underlying DSP [3], [12].

We therefore aimed to determine whether the clinical characteristics, electrodiagnostic classification of nerve injury and treatment response differed in diabetes and non-diabetes patients diagnosed with CIDP; and whether these characteristics could be useful to differentiate the two entities.

## Methods

### Subjects

CIDP subjects attending the Neuromuscular clinic for management of their immune-mediated polyneuropathy between 1997 and 2013 were evaluated for this study. Our current study involved the extraction of demographic data, clinical history, physical examination, laboratory test results, and electrophysiologic data from previously coded charts of CIDP patients. The Research Ethics Board of the University Health Network approved the current study protocol, based on chart review and collection of de-identified data.

All subjects were ≥18 years of age and had a diagnosis of CIDP. All subjects with a confirmed diagnosis of either type 1 or type 2 diabetes mellitus (DM) and CIDP were included in this study (n = 67). The duration of diabetes mellitus was taken from the time when the patients first developed symptoms or from the time that they were diagnosed as having diabetes by their physician, if asymptomatic. CIDP-DM patients were selected from the full CIDP cohort (n = 1950) and were matched based on sex and age to the CIDP+DM cohort, using 1∶1 matching. DM was diagnosed according to the American Association of Diabetes criteria based on one of four abnormalities: haemoglobin A1c, fasting plasma glucose, random elevated glucose with symptoms, or abnormal oral glucose tolerance test [13]. CIDP was diagnosed based on the clinical presentation, as judged by a neuromuscular expert (VB), and the presence of demyelination on NCS as per the Koski criteria [2]. CIDP+DM patients may also have a diagnosis of DSP according to the following criteria: at least one abnormal sural NCS result, one abnormal peroneal NCS result, and at least one neuropathic sign or symptom [1], [14], [15]. This study excluded patients with proximal diabetic radiculoplexopathies based on clinical presentation; i.e.: patients presenting with asymmetrical, acute proximal leg pain followed by weakness, as the pain settled. The duration of polyneuropathy was taken as the time from when the patient first developed symptoms to their evaluation in the Neuromuscular clinic.

CIDP patients, with or without DM, were also classified as responders (R) (n = 46) or non-responders (n = NR) (54) based on clinical response to treatment assessed by a combination of patient and physician evaluations in the 100 patients who were treated with immunotherapies. R subjects were those who stabilized after declining progressively, or improved after treatment, and NR subjects either worsened or did not change after treatment based on their clinical evaluation.

Subjects were evaluated for neuropathy by neurological examination, the 19-point Toronto Clinical Neuropathy Score (TCNS), vibration perception thresholds (VPT), and sural, peroneal and tibial NCS [2], [16]. NCS were performed using the Sierra Wave instrument (Cadwell Laboratories Inc., Kennewick, WA, USA). Age- and height-adjusted NCS reference values were used, according to the standards of the Toronto General Hospital (University Health Network) electrophysiology laboratory. Limb temperature was measured prior to nerve conduction studies, and if required, warming was performed to ensure a surface temperature of ≥32.0°C in the hands and ≥31.0°C in the feet.

Sural, peroneal and tibial NCS were performed using surface stimulating and recording techniques according to the standards of the Canadian Society of Clinical Neurophysiology and the American Association of Neuromuscular and Electrodiagnostic Medicine [17], [18]. The Electromyography Instrument calculated latencies, amplitudes and conduction velocities automatically. Peroneal and tibial nerve motor amplitudes were measured as baseline to peak for the compound muscle action potential [CMAP] at the ankle and the knee, and for baseline to negative peak for the sural sensory nerve action potential [SNAP] amplitude, or from the positive peak (if present) to the negative peak. The sural nerve latency was measured at the onset from the initial deflection from baseline. The F wave latency was determined as the minimum reproducible latency obtained after 10 supramaximal stimuli were applied to the tibial and peroneal nerves at the ankle.

Vibration perception thresholds were measured using the Neurothesiometer (Howell Scientific, London, UK) using the method of limits. Three tests were performed on the dorsum of each first toe proximal to the nail bed and a mean of the 3 responses was calculated. A “null” test was randomly inserted into the test sequence to ensure that the patient was reporting vibrating sensation accurately.

### Statistical Analysis

Statistical analysis was performed using JMP (version 9.0.2 for Macintosh, from SAS). Demographic data were expressed as means ± standard deviation (SD) for normally distributed data, or median and interquartile range [IQR] for data not normally distributed. Differences in categorical variables were assessed using the χ^2^-test, while differences in continuous variables were assessed using the ANOVA, or the Kruskal-Wallis test for non-parametric data. Given the multiple comparisons, for each set of analyses significance was adjusted with Bonferroni correction, using a baseline p-value of 0.05, divided by the number of comparisons done.

## Results

The demographic data of the 134 CIDP subjects categorized with diabetes (CIDP+DM, n = 67) or without diabetes (CIDP-DM, n = 67) are shown in Table 1. The 134 CIDP subjects had a mean age of 65.8±13.5 years and mean haemoglobin A_1c_ (HbA_1c_) of 7.1±1.9%. CIDP+DM subjects had higher systolic blood pressures (p = 0.04), higher lower limb vibration potential thresholds (VPT) (right p = 0.004, left p = 0.01), and more abnormal sensory testing as indicated by Toronto Clinical Neuropathy Score (TCNS) (p = 0.0009) than CIDP-DM subjects. CIDP+DM subjects also had weaker hip flexors (p = 0.05) and weaker quadriceps (p = 0.03), and more abnormality of gait (p = 0.03) compared to CIDP-DM patients. CIDP+DM subjects had higher HbA_1c_ values (7.7±2.0%) than CIDP-DM patients (5.6±0.4%, p<0.001) as expected, and a higher prevalence of retinopathy, nephropathy and hypertension (p = 0.001, p = 0.0007, p = 0.0005).

**Table 1:** Clinical and electrodiagnostic features of 67 CIDP-DM and 67 CIDP+DM subjects.

	CIDP-DM and CIDP+DM subjects (n = 134)		
	CIDP-DM	CIDP+DM	P value
n	67	67	
Age (years)*	66.5±13.4	65.1±13.7	0.55
Male sex, n (%)	48 (72%)	46 (69%)	0.71
BMI (kg/m^2^)	27.8±4.7	27.7±6.0	0.99
Type 2 DM, n (%)		65 (97%)	
Duration DM (years)		16.5±13.5	
Duration PNP (years)	9.73±7.4	9.93±8.5	0.89
Systolic blood pressure (mmHg)	132.6±16.6	140.8±21.8	0.04
Diastolic blood pressure (mmHg)	79.8±10.2	81.5±12.8	0.45
VPT upper right	6.3±5.3	7.6±4.6	0.15
VPT upper left	6.2±5.3	7.6±5.2	0.15
VPT lower right	24.1±13.9	31.4±13.4	0.004
VPT lower left	23.9±13.6	30.2±12.9	0.01
TCNS, Median [IQR]†	13 [8], [16]	13 [9], [16]	0.45
Symptoms, Median [IQR]	4 [3], [5]	4 [3], [5]	0.30
Sensory, Median [IQR]	4 [2], [4]	4 [3], [5]	0.0009^¥^
DTR, Median [IQR]	6 [4], [8]	6 [4], [8]	0.42
Retinopathy, n (%)	1 (1%)	11 (16%)	0.001
Nephropathy, n (%)	0 (0%)	8 (12%)	0.0007^¥^
Hypertension, n (%)	22 (33%)	42 (63%)	0.0005^¥^
Hyperlipidemia, n (%)	25 (37%)	29 (43%)	0.48
HbA_1c_, %‡	5.6±0.4	7.7±2.0	<0.0001^¥^
Nerve conduction parameters			
Sural nerve amplitude potential (µV)	6.6±6.0 (1.3–30.2)§	2.4±3.0 (0–14.2)	<0.0001^¥^
Sural nerve distal latency (ms)	3.4±0.5 (1.5–4.5)	3.6±0.6 (2.1–5.1)	0.098
Sural nerve conduction velocity (m/s)	41.0±5.3 (31.0–56.0)	38.6±5.4 (27.0–57.0)	0.04
Peroneal nerve amplitude potential (mV) - ankle	2.7±2.8 (0–12.8)	2.0±2.4 (0–11.6)	0.15
Peroneal nerve amplitude potential (mV) - knee	2.2±2.5 (0–11.6)	1.8±2.4 (0–13.1)	0.41
Peroneal nerve distal latency (ms)	8.0±5.0 (3.2–32.8)	6.0±1.4 (3.2–9.5)	0.003
Peroneal nerve conduction velocity (m/s) – fibular head	33.8±7.3 (15.0–47.0)	32.4±6.4 (17.0–45.0)	0.28
Peroneal nerve conduction velocity (m/s) – popliteal fossa	35.8±7.3 (18.0–53.0)	32.4±6.4 (22.0–47.0)	0.35
Peroneal nerve F-wave (ms)	54.3±26.7 (0–95.7)	59.2±16.1 (0–80.8)	0.44
Conduction block (%)	13.2±23.3	9.77±44.2	0.61
Tibial nerve amplitude (mV)	5.3±6.1 (0–24.2)	5.6±5.5 (0.3–23.3)	0.78
Tibial nerve amplitude (mV) – popliteal fossa	3.8±4.6 (0–18.3)	3.9±3.8 (0.1–16.4)	0.92
Tibial nerve distal latency (ms)	7.0±3.2 (3.9–18.5)	5.4±1.2 (3.3–8.5)	0.003
Tibial nerve distal latency (ms) – popliteal fossa	18.3±5.5 (0.9–32.1)	17.0±3.2 (10.2–23.8)	0.19
Tibial nerve conduction velocity (m/s)	34.2±6.7 (20–53)	35.0±6.1 (22–49)	0.59
Tibial nerve F-wave (ms)	66.1±5.6 (53.8–77.0)	64.0±8.5 (43.1–80.6)	0.29

Data are means ± SD unless otherwise indicated.

Differences in categorical variables were assessed in three-group comparisons using the χ^2^-test, while differences in continuous variables were assessed using the ANOVA except in the case of TCNS in which the Kruskal-Wallis test was applied.

*The mean age for the 134 CIDP-DM and CIDP+DM subjects was 65.8±13.5 years.

† Toronto Clinical Neuropathy Score (TCNS) is a clinical indicator of the severity of neuropathy, with 0–4, 5–8, and ≥9 indicating no, mild, and moderate to severe neuropathy. Values less than 5 are normal. For the deep tendon reflex (DTR) segment of the TCNS, the normal value is 0. For TCNS, median and interquartile range (IQR) are shown and IQR are compared.

‡ The mean HbA_1c_, indicating the percentage of haemoglobin A_1c_, for 71 of the 134 CIDP-DM and CIDP+DM subjects was 7.1±1.9%.

§ Below the NCS mean parameter values, the range for that parameter is shown in brackets.

BMI = body mass index; DM = diabetes mellitus; PNP = polyneuropathy; VPT = vibration perception threshold; DTR = deep tendon reflexes of the lower limb; NR = non-recordable.

**¥ Bonferroni corrected p-value for significance = 0.001.**

On NCS, CIDP+DM subjects had shorter peroneal distal motor latencies (6.0±1.4 ms vs 8.0±5.0 ms, p = 0.003), shorter tibial distal motor latencies (5.4±1.2 ms vs 7.0±3.2 ms, p = 0.003), lower sural sensory nerve action potential amplitudes (2.4±3.0 µV vs 6.6±6.0 µV, p<0.0001) and slower sural conduction velocities (38.6±5.4 m/s vs 41.0±5.3 m/s, p = 0.04) than CIDP-DM subjects. Details of immunomodulatory and immunsuppressive treatment are shown in Table 2. CIDP-DM subjects received more treatment (93% vs57%, p = <0.0001) than CIDP+DM subjects, despite no significant difference in clinical response to any treatment (p = 0.71). Howeever, CIDP-DM patients had a higher response rate to IVIG treatment than CIDP+DM, although the response rate is higher than previously observed [19].

**Table 2:** Treatment details of 67 CIDP-DM and 67 CIDP+DM subjects.

	CIDP-DM and CIDP+DM subjects (n = 134)		
	CIDP-DM	CIDP+DM	P value
n	67	67	
Response to treatment (n = 100)			0.71
Non-responders, n (%)	29 (45%)	17 (49%)	
Responders, n (%)	36 (55%)	18 (51%)	
Treatment Provided, n (%)	62 (93%)	36 (57%)	<0.0001^¥^
IVIG, n (%)	58 (87%)	33 (52%)	<0.0001^¥^
Prednisone, n (%)	44 (67%)	12 (19%)	<0.0001^¥^
PLEX, n (%)	10 (15%)	3 (5%)	0.04
azathioprine, n (%)	36 (55%)	7 (11%)	<0.0001^¥^
mycophenolate mofetil, n (%)	9 (14%)	6 (10%)	0.46
Loading dose IVIG (2 G/kg)	1.86±0.4	1.97±0.4	0.23
Number of IVIG treatments	22.4±39.6 (1–200)*	7.02±12.2 (0–60)	0.02
Response to IVIG treatment, n (%)	46 (84%)	18 (56%)	0.006
Number of PLEX treatments	1.4±0.9 (1–3)	4.7±0.6 (4–5)	0.0002^¥^
Response to PLEX treatment, n (%)	9 (82%)	2 (67%)	0.59
Clinical Status (n = 100)			0.13
Worse, n (%)	16 (25%)	4 (11%)	
No change, n (%)	13 (20%)	13 (37%)	
Stablized, n (%)	21 (32%)	8 (23%)	
Improved, n (%)	15 (23%)	10 (29%)	
NCS after treatment (n = 93)			0.85
Worse, n (%)	8 (14%)	6 (18%)	
Stable, n (%)	48 (81%)	26 (76%)	
Improved, n (%)	3 (5%)	2 (6%)	

Data are means ± SD unless otherwise indicated.

Differences in categorical variables were assessed in three-group comparisons using the χ^2^-test, while differences in continuous variables were assessed using the ANOVA.

*Below the number of treatment mean values, the range for that treatment number is shown in brackets.

CIDP = chronic immune demyelinating polyneuropathy; DM = diabetes mellitus; IVIG = intravenous immunoglobulin; PLEX = plasma exchange; NCS = nerve conduction studies.

**¥ Bonferroni corrected p-value for significance = 0.003.**

Of the 134 CIDP subjects, with or without DM, 100 received treatment for CIDP and the clinical and electrodiagnostic features of the 46 NR and 54 R subjects are shown in Table 3. When looking at response to any treatment, R subjects had a much shorter duration of polyneuropathy than NR subjects (8.0±6.0 y vs 11.9±7.6 y, p = 0.004).

**Table 3:** Clinical and electrodiagnostic features of 46 Non-Responders (NR) and 54 Responders (R) to treatment for CIDP.

	NR and R CIDP subjects (n = 100)		
	NR	R	P value
n	46	54	
Age (years)*	67.4±12.4	63.5±14.6	0.16
Male sex, n (%)	30 (65%)	39 (72%)	0.45
BMI (kg/m^2^)	28.3±4.7	28.4±6.1	0.97
Duration DM (years)	18.4±14.0	14.1±7.4	0.26
Duration PNP (years)	11.9±7.6	8.0±6.0	0.004
Systolic blood pressure (mmHg)	136.8±21.5	133.4±19.3	0.47
TCNS, Median [IQR]†	13.5 [11], [15]	13 [7.75,16]	0.41
Nerve conduction parameters			
Sural nerve amplitude potential (µV)	3.9±5.9 (0–30.2)§	5.5±5.0 (0–18.8)	0.18
Peroneal nerve amplitude potential (mV) - ankle	2.1±2.2 (0–7.7)	2.5±2.9 (0–12.8)	0.58
Peroneal nerve conduction velocity (m/s) – fibular head	33.5±7.8 (15.0–46.0)	32.6±7.3 (15.0–47.0)	0.59
Conduction block (%)	16.7±17.3	13.3±31.5	0.57

Data are means ± SD unless otherwise indicated.

Differences in categorical variables were assessed in three-group comparisons using the χ^2^-test, while differences in continuous variables were assessed using the ANOVA except in the case of TCNS in which the Kruskal-Wallis test was applied.

*The mean age for the 100 NR and R CIDP subjects was 65.3±13.7 years.

† Toronto Clinical Neuropathy Score (TCNS) is a clinical indicator of the severity of neuropathy, with 0–4, 5–8, and ≥9 indicating no, mild, and moderate to severe neuropathy. Values less than 5 are normal.

§ Below the NCS mean parameter values, the range for that parameter is shown in brackets.

BMI = body mass index; DM = diabetes mellitus; PNP = polyneuropathy.

**¥ Bonferroni corrected p-value for significance = 0.004.**

## Discussion

We examined a matched cohort of CIDP patients with and without diabetes to compare their clinical characteristics, electrodiagnostic classification of nerve injury and response to treatment. This report is the largest cohort so examined. We observed that CIDP+DM patients had a different clinical profile when compared to patients with CIDP-DM. Specifically, CIDP+DM patients had more severe neuropathy and a similar treatment response rate compared with CIDP-DM patients as observed by Gorson [5], but were less likely to receive treatment. This occurred despite more evidence of large fibre dysfunction, including higher rates of proximal weakness and ataxia. The only factor that was associated to treatment response in these patients was duration of polyneuropathy, but not the presence or duration of diabetes. Although the response rate to treatment with IVIG tended to be higher in CIDP-DM patients than in CIDP+DM patients, the response rate in the CIDP-DM group is higher than observed in prior studies [19] and may reflect sampling error. The overall response rate to all treatments was no different between the 2 populations. The main objective of this study was to compare the clinical characteristics and treatment patterns of CIDP+DM and CIDP-DM patients, and not to look at predictors of response to treatment in CIDP patients. This is why patients were matched based on the presence and absence of DM and not based on their responder status. The latter would be a case-control study and might better answer the specific question of how DM status affects response to treatment.

Existing criteria for the diagnosis of CIDP are highly specific [3], [20] and may lack the necessary sensitivity to diagnose immunologically-based demyelination in diabetes patients, leading to under-treatment in CIDP+DM patients, as observed in the current study. In part, the under-treatment may be due to the difficulty differentiating the impairments due to CIDP from those due to DSP because of the overlap in clinical and electrophysiological characteristics of CIDP and DSP. In these patients, previous diabetes-related nerve injury may mask demyelinating changes caused by novel immune-mediated nerve injury. Thus, as the study results suggest, we may fail to adequately treat CIDP in diabetes patients, a finding we did not expect at the start of this study, and this finding underscores the need for a serum or tissue biomarker.

We have previously reported that diabetes patients demonstrate electrophysiological findings atypical for classic DSP, although insufficient to make a diagnosis of CIDP using existing criteria [4]. For example, although a small reduction in conduction velocity can be expected in DSP, more robust slowing in conduction velocity, not meeting the range of rigorous criteria for CIDP, is evidence for demyelination in the context of DSP [4]. This demyelination is related to differing clinical features, namely, more impaired glycemic control (mean HbA1C of 9.6%±2.4%) in type 1 diabetes patients compared to the axonal form of DSP (mean HbA1C of 7.5%±1.1). In this setting, CIDP is less likely than poorly controlled diabetes and the treatment needs to be tailored accordingly. We have also observed previously that patients with the combination of CIDP and diabetes have better glycemic control, more severe neuropathy and a shorter duration of diabetes when compared to those who have the demyelinating form of DSP (mean HbA1C of 7.7%±2.0% vs 8.9%±2.3%) [21]. So, relatively good glycemic control with more severe neuropathy and demyelinating features in a diabetes patient should trigger the diagnostic consideration of CIDP with concomitant implications for therapy. The current study results indicate that this is not always the case.

Limitations of the current study are that this is a retrospective review. Although outcomes are based on clinical evaluation by both the physician and patient, rigorous scales such as the inflammatory cause and treatment (INCAT) scale, the Rasch-built Overall Disability Scale (RODS) and the Overall Neuropathy Limitation Scale (ONLS) were not available for most patients when this study was done, although current practice has changed[22]–[24]. The use of such scales in future studies would help define responder status in a more standardized fashion. Since there are no serum biomarkers to make a definitive diagnosis of CIDP and given that demyelination on NCS is a non-specific finding, misclassification of subjects is a potential error. Sural nerve matrix mtelloproteinase-9 is a possible tissue biomarker of CIDP in patients with diabetes, but sural nerve biopsy is invasive and unlikely to be performed routinely in all patients [25]. We acknowledge the challenges in differentiating CIDP+DM and proximal forms of diabetic neuropathy, such as diabetic lumbosacral plexoradiculoneuropathy, which may have lead to some misclassification of cases in our study. However, this problem was at least partially mitigated by design in that those patients presenting with typical clinical features of proximal diabetic neuropathies were excluded. Also, we observed a selection bias against treatment in CIDP+DM patients. If all CIDP+DM were treated then the percentage of responders in this cohort might be less than observed and differ from those with CIDP-DM.

The differentiation of CIDP in patients with and without diabetes is important due to the implications for therapy and prognosis. This study shows that CIDP+DM patients have more severe clinical and electrophysiological neuropathy than CIDP-DM patients, but that they do not receive the same treatment despite having similar treatment response rates. We know that severity of neuropathy does not prevent response to treatment as shown by the ICE study results [19]. Therefore, it is crucial to raise awareness of the presence of CIDP in diabetes patients so that they can be offered appropriate therapy rather than being diagnosed as having an irreversible neuropathy; namely, DSP. Our results show differences in cohorts of CIDP patients with and without diabetes, but do not provide cut-off values for differentiating between these 2 diagnoses in individual patients. Future work directed towards finding specific diagnostic cut-off values, and a specific serum biomarker for the diagnosis of immune-mediated polyneuropathies in patients with diabetes has major implications for improved care of these patients.
